# Extraction of parameters of a stochastic integrate-and-fire model with adaptation from voltage recordings

**DOI:** 10.1007/s00422-024-01000-2

**Published:** 2024-12-30

**Authors:** Lilli Kiessling, Benjamin Lindner

**Affiliations:** 1 Bernstein Center for Computational Neuroscience Berlin, Philippstr. 13, Haus 2, 10115 Berlin, Germany; 2 Physics Department of Technische, Universit Berlin, Hardenbergstr. 36, 10623 Berlin, Germany; 3 Physics Department, Humboldt University Berlin, Newtonstr. 15, 12489 Berlin, Germany

**Keywords:** Stochastic spiking, Integrate-and-fire model, Spike-frequency adaptation, Parameter extraction for neural models

## Abstract

Integrate-and-fire models are an important class of phenomenological neuronal models that are frequently used in computational studies of single neural activity, population activity, and recurrent neural networks. If these models are used to understand and interpret electrophysiological data, it is important to reliably estimate the values of the model’s parameters. However, there are no standard methods for the parameter estimation of Integrate-and-fire models. Here, we identify the model parameters of an adaptive integrate-and-fire neuron with temporally correlated noise by analyzing membrane potential and spike trains in response to a current step. Explicit formulas for the parameters are analytically derived by stationary and time-dependent ensemble averaging of the model dynamics. Specifically, we give mathematical expressions for the adaptation time constant, the adaptation strength, the membrane time constant, and the mean constant input current. These theoretical predictions are validated by numerical simulations for a broad range of system parameters. Importantly, we demonstrate that parameters can be extracted by using only a modest number of trials. This is particularly encouraging, as the number of trials in experimental settings is often limited. Hence, our formulas may be useful for the extraction of effective parameters from neurophysiological data obtained from standard current-step experiments.

## Introduction

Integrate-and-fire (IF) neuron models are widely used in theoretical studies of neural dynamics (see e.g. Johannesma 1968; Knight 1972; Treves 1993; Campbell et al. 1999; Brunel 2000; Brunel et al. 2001; Lindner et al. 2005; de la Rocha et al. 2007; Litwin-Kumar and Doiron 2012; Lindner 2022) and reviews (Holden 1976; Ricciardi 1977; Tuckwell 1989; Burkitt 2006a, b). These models simplify the complex properties of neurons into a manageable framework, making it possible to analyze spontaneous neural activity and predict neural responses to time-dependent stimuli. Although basic in nature, IF models capture the timing of neuronal spikes effectively, which is crucial for understanding how neurons communicate and process information (Gerstner and Naud 2009).

The leaky integrate-and-fire (LIF) model (Lapicque 1907; Stein 1967; Tuckwell 1988) combines input integration with a fire-and-reset rule. It was previously shown, that including mechanisms for adaptation is important to capture neural spike process properly (Benda and Herz 2003; Brette and Gerstner 2005). Another important addition is the incorporation of a noise source to account for the notorious stochasticity of spike generation in many situations. Often, the noise that may stem from channel fluctuations or from synaptic inputs is low-pass filtered in time-due to slow channel kinetics (Schwalger et al. 2010; Fisch et al. 2012) and synaptic dynamics (Brunel and Sergi 1998; Moreno-Bote and Parga 2010), respectively. A standard choice of a model with Gaussian low-pass filtered noise is the stochastic Ornstein-Uhlenbeck process (originally introduced to model the velocity of a Brownian particle (Uhlenbeck and Ornstein 1930). Gaussian statistics arise in many situations when an abundance of nearly independent inputs add up - these can be currents through many ion channels or the inputs at many synapses. We mention in passing that other relevant noise statistics in neurons are shot noise (when the spike character of synaptic input cannot be neglected, see e.g. Richardson and Swarbrick 2010; Droste and Lindner 2017; Richardson 2024) or dichotomous noise (when up/down states from a surrounding network dominate the fluctuation input, see e.g. Droste and Lindner 2014; Mankin and Lumi 2016).

Accurately identifying model parameters that reflect experimental data is essential for the utility of these models in experimental and theoretical studies (Paninski et al. 2003; Huys et al. 2006; Rossant et al. 2011; Iolov et al. 2017; Ladenbauer et al. 2019; Friedrich et al. 2014). Traditional methods for parameter estimation in IF models often rely on numerical fitting (Friedrich et al. 2014; Teeter et al. 2018). In some experiments in vitro, a noisy current (in the form of a computer-generated Ornstein-Uhlenbeck process) is injected into the cell, which allows to extract subthreshold nonlinearities and their parameters directly; see e.g. the pioneering studies by Badel et al. (2008a, 2008b). Other studies (Vilela and Lindner 2009a, b) have provided relations of the firing statistics of simple IF models with white Gaussian noise, specifically their firing rate and coefficient of variation of the interspike interval (ISI) to the input parameters (base current and noise intensity). Because the neural spiking process is inherently nonlinear, and not all relevant variables are also observable (adaptation currents are difficult to access), the estimation of parameters of spiking neuron models based on experimental data remains a difficult task.

In our study, we introduce a new analytical method that derives essential parameters of the adaptive leaky integrate-and-fire model with an (unknown) low-pass filtered Gaussian noise. We assume that we know the response of the membrane voltage to a current-step for a sufficiently large number of trials. The method provides the adaptation time constant, adaptation strength, membrane time constant, and mean input current. Importantly, it does not require explicit knowledge of the time course or characteristics of the intrinsic noise, making it applicable to a wide range of experimental conditions. This approach can potentially facilitate the classification of neuron types (Teeter et al. 2018) and the exploration of fluctuation-response relationships in experimental settings (Lindner 2022, 2002b; Puttkammer and Lindner 2024).

This paper is structured as follows: we begin by describing the adaptive integrate-and-fire model with Ornstein-Uhlenbeck noise, explain the new method for extracting parameters, validate this method with numerical simulations, and, finally, briefly summarize our finding and give an outlook to possible extensions of the method.

## Model and measures

The adaptive integrate-and-fire model with exponentially correlated (colored) noise describes the dynamics of a neuron using a system of stochastic differential equations

1a $$\begin{aligned} \tau _m \frac{d}{dt}v&= -v + \mu -a + \eta - \tau _m (v_T - v_R) x(t) + s \end{aligned}$$

1b $$\begin{aligned} \tau _a \frac{d}{dt}a&= - a + \Delta _a \tau _a x(t) \end{aligned}$$

1c $$\begin{aligned} \tau _\eta \frac{d}{dt} \eta&= -\eta + \sqrt{2 \sigma ^2 \tau _\eta } \xi (t). \end{aligned}$$

Here, *v*(*t*) represents the dynamics of the membrane potential, *a*(*t*) is the adaptation variable, and $\eta (t)$ corresponds to the correlated noise. Furthermore, *s*(*t*) is an external perturbation (here a step current), $\mu$ is the effective intrinsic mean input current, and $x(t) = \sum _i \delta (t - t_i)$ is the spike train, in which we sum over the spike times $t_i$. The latter are determined by the fire-and-reset rule that is applied to the first Eq. (1a) above: Whenever the membrane voltage *v*(*t*) exceeds the threshold $v_T$, a spike time $t_i$ is registered and the membrane voltage is reset to $v_R$. Note that we have already formally incorporated the reset by the term $- \tau _m (v_T - v_R) x(t)$ in the first Eq. (1a) and that the model, despite its apparent linearity in the variables $v(t), a(t), \eta (t)$, is highly nonlinear due to the fire-and-reset rule.

The spike train affects the adaptation variable in the second Eq. (1b) via the term $\tau _a \Delta _a x(t)$. The jump of the adaptation variable by $\Delta _a$ with each generated spike is usually referred to as a *spike-triggered adaptation* and a form of negative feedback. Put differently, the negative-feedback effect of *a*(*t*) in the voltage equation leads to a downregulation of the spiking if the activity is high.

**Fig. 1:**
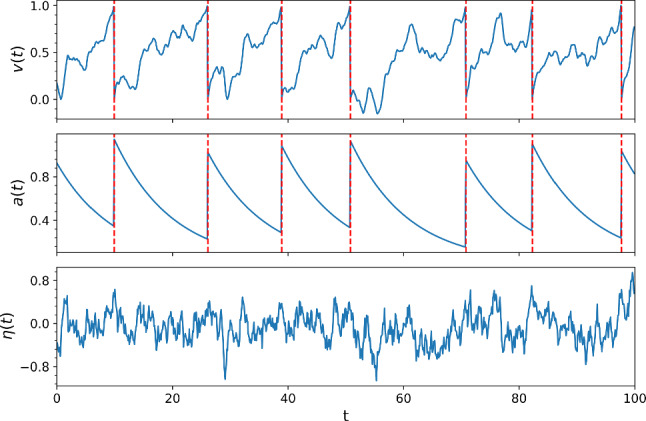
Stochastic integrate-and-fire neuron with $\mu = 1.2, \tau _m = 1, \tau _a = 10, \Delta _a = 0.8, \tau _\eta = 1, \sigma ^2 = 0.1, s = 2.0, \Delta t=0.01$

The third Eq. (1c) describes an Ornstein-Uhlenbeck process, which serves here (via a Markovian embedding) as a source of colored noise with vanishing mean $\left\langle \eta (t) \right\rangle =0$, variance $\sigma ^2$, correlation-time $\tau _\eta$, and an exponential correlation function $\langle \eta (t)\eta (t+\tau ) \rangle = \sigma ^2 \exp [- |\tau |/\tau _\eta ]$. This process can be regarded as a low-pass filtered version of the Gaussian white noise $\xi (t)$ (correlation function $\langle \xi (t) \xi (t + \tau ) \rangle = \delta (\tau )$), emerging from a synaptic filtering of massive Poissonian input spike trains in the diffusion approximation (Richardson and Gerstner 2005). Figure 1 depicts the time courses of the membrane potential, the adaptation variable, and the Ornstein-Uhlenbeck noise for one trial under stationary conditions ($s(t) \equiv 0$).

The model contains a number of parameters: The effective mean input current $\mu$, the membrane time constant $\tau _m$, the adaptation time constant $\tau _a$, and the adaptation step $\Delta _a$. All these parameters, $\mu$, $\tau _m$, $\tau _a$ and $\Delta _a$, have to be determined if the model is supposed to describe the activity of an individual real neuron. The noise parameters $\sigma$ and $\tau _\eta$ are not extracted as they do not influence the relations among the different averaged observables, which are used for the inference of the parameters (although the strength and type of noise will affect the averaged observables themselves). Typically, for the model class of integrate-and-fire neurons, numerical methods of parameter fitting are used (Teeter et al. 2018). If a time-dependent stimulus is known, for instance, as a frozen-noise stimulus $\eta (t)$, more information about the underlying voltage dynamics can be extracted directly from pyramidal cells (Badel et al. 2008b) and fast-spiking interneurons (Badel et al. 2008a). Here, we do not assume that the time course of the colored intrinsic noise is known, but that we know trials of voltage traces in response to a current step $s(t)=\varepsilon \Theta (t-t_\varepsilon )$. We will show that all unknown parameters can be obtained from explicit formulas, and we will test these formulas for broad ranges of parameter values. We exclusively use data from model simulations to test the method. To apply the method to experimental data is beyond the scope of our current study, but an exciting problem for future investigations.

The model Eqs. (1a – 1c) are integrated using the Euler-Maruyama method with a time step of $\Delta t= 10^{-5}$. To reduce the amount of data, we resample them at a coarse-grained resolution of $\Delta t_s = 10^2 \cdot \Delta t$. This time step is also used for the numerical evaluation of the Laplace transforms that play an important role in our theory (see below). In the first trial, we initialize the values of *v*(*t*) and *a*(*t*) at zero, leading to a transient phase before reaching a steady state. In subsequent simulation trials, the last values from the previous simulation before stimulus onset are used as the new initial values, in this way effectively sampling the steady state of the system.

The trials from our simulations can be averaged to obtain estimates of the mean voltage $\langle v(t) \rangle$ and the mean adaptation $\left\langle a(t) \right\rangle$ ($N_{\text {trials}}=1000$ are used if not indicated otherwise). We note that only the voltage *v*(*t*) and the spike train *x*(*t*) are directly accessible in the experiment, whereas the adaptation *a*(*t*) is a hidden variable. For this reason, we will seek to eliminate $\left\langle a(t) \right\rangle$ from subsequent equations. We can estimate the instantaneous firing rate by Gerstner et al. (2014)

2 $$\begin{aligned} r(t) = \langle x(t) \rangle \approx \frac{\sum _{k=1}^{N_{\text {trials}}} \sum _j n_k (t, t+ \Delta t_s)}{\Delta t_s \cdot N_{\text {trials}}}, \end{aligned}$$

where $n_k(t, t+ \Delta t_s)$ is the count of threshold crossings in the time interval $[t, t+\Delta t_s]$. If the system is in a stationary state, we can additionally average over time and use a mixed ensemble-time average. We note that in an experimental situation, the trials could be taken from a long single experiment in which the injected current is switched back and forth between two values. In order for the trials to be statistically independent, we have to make sure that the time window of the single trial is much longer than the adaptation time constant $\tau _a$ and the correlation time constant of the noise $\tau _\eta$.

## Calculation of mean values in steady state

Here, we analyze the steady state of the adaptive integrate-and-fire model under stationary conditions, i.e. with a constant input current of either $\mu$ or $\mu + \varepsilon$. Stationarity implies that all averaged time derivatives and the average noise are zero, $\left\langle dv/dt \right\rangle =0, \left\langle da/dt \right\rangle =0, \left\langle \eta (t) \right\rangle =0$. An ensemble average of Eqs. 1a and 1b for $s(t)=0$ results in

3a $$\begin{aligned} \langle v \rangle _0&= \mu - \langle a \rangle _0 - \tau _m (v_T - v_R) r_0 \end{aligned}$$

3b $$\begin{aligned} \langle a \rangle _0&= \Delta _a \tau _a r_0, \end{aligned}$$

which corresponds to the initial steady state, i.e. $\langle v \rangle _0$, $\langle a \rangle _0$, and $r_0$ denote the steady-state averages of voltage, adaptation variable, and spike train, respectively, before the stimulus onset.

After the stimulus is applied for a sufficiently long duration, a new steady state is reached and a similar calculation yields the new steady-state averages under a constant current of $\mu + \varepsilon$

4a $$\begin{aligned} \langle v \rangle _\varepsilon&= \mu + \varepsilon - \langle a \rangle _\varepsilon - \tau _m (v_T - v_R) r_\varepsilon \end{aligned}$$

4b $$\begin{aligned} \langle a \rangle _\varepsilon&= \Delta _a \tau _a r_\varepsilon . \end{aligned}$$

**Fig. 2:**
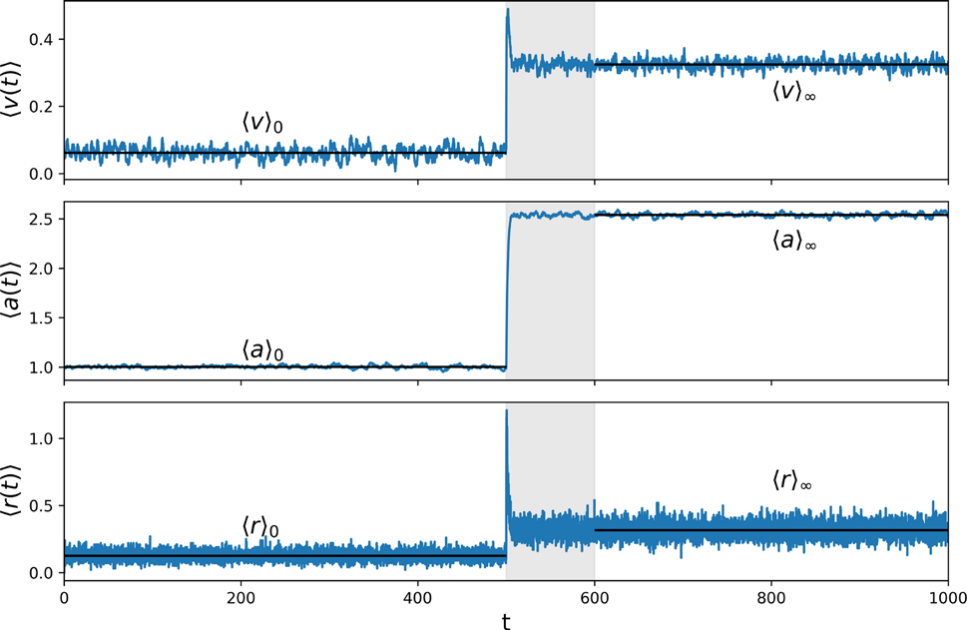
Steady-state and time-dependent averages $\langle v(t) \rangle , \langle a(t) \rangle , \langle r(t) \rangle$ (light gray area) when the stimulation current is turned on $\varepsilon = 2.0$ at $t_\varepsilon = 500$. $N_{\text {trials}}=1000$ with $\mu = 1.5, \tau _m = 1, \tau _a = 10, \Delta _a = 0.8, \tau _\eta = 1, \sigma ^2 = 1$. Integration step $\Delta t = 0.001$; time series stored with $\Delta t_s = 0.1$

Fig. 2 illustrates the steady-state values along with the time-dependent features of the dynamics that we have not described yet. Here, we have used a very conservative (long) length of the window. It is visible that we could easily choose a shorter window without violating the assumption of independent trials, while still capturing the necessary dynamics. Using shorter trials would imply a larger number of trials obtained from one long experiment.

The steady state Eqs. (3a– 4b) obtained so far relate measurable mean values $\left\langle v \right\rangle , \left\langle a \right\rangle , r$ with the unknown parameters $\tau _m, \mu , \tau _a, \Delta _a$. For the differences in the steady-state averages before and long after the onset of the stimulation we obtain

5a $$\begin{aligned} \left\langle \delta v \right\rangle&= \langle v \rangle _\varepsilon - \langle v \rangle _0 = \varepsilon - \left\langle \delta a \right\rangle - \tau _m (v_T - v_R) \delta r \end{aligned}$$

5b $$\begin{aligned} \left\langle \delta a \right\rangle&= \langle a \rangle _\varepsilon - \langle a \rangle _0 = \Delta _a \tau _a \delta r, \end{aligned}$$

where $\left\langle \delta v \right\rangle$ and $\left\langle \delta a \right\rangle$ represent the mean changes in membrane potential and adaptation, respectively, and $\delta r = r_\varepsilon - r_0$ denotes the change in firing rate.

These equations allow to express the adaptation difference $\left\langle \delta a \right\rangle$ in terms of the changes in membrane potential $\left\langle \delta v \right\rangle$ and firing rate $\delta r$. Equation (5a) leads to the relationship

6 $$\begin{aligned} \left\langle \delta a \right\rangle = \varepsilon - \left\langle \delta v \right\rangle - \tau _m (v_T - v_R) \delta r , \end{aligned}$$

which also incorporates the model parameter $\tau _m$. Furthermore, Eq. (5b) allows for deducing a reciprocal relationship for the adaptation parameters $\Delta _a$ and $\tau _a$

7 $$\begin{aligned} \Delta _a = \frac{\left\langle \delta a \right\rangle }{\tau _a \delta r} = \frac{\varepsilon - \left\langle \delta v \right\rangle - \tau _m (v_T - v_R) \delta r}{\tau _a \delta r}. \end{aligned}$$

The derived relations, Eqs. (6) and (7), do not suffice to entirely determine the unknown parameters; we will need additional information on the system, which is obtained from the temporal behavior, an analysis that is presented in the next section.

## Transient analysis

With stimulus onset the system exhibits transient behavior in membrane potential, adaptation variable, and firing rate. The equations capturing the temporal evolution of the mean membrane potential and mean adaptation are

8a $$\begin{aligned} \tau _m \frac{d}{dt} \langle v(t) \rangle&= - \langle v(t) \rangle + \mu + \varepsilon \nonumber \\&\quad - \langle a(t) \rangle - \tau _m (v_T - v_R) r(t), \end{aligned}$$

8b $$\begin{aligned} \tau _a \frac{d}{dt} \langle a(t) \rangle&= - \langle a(t) \rangle + \Delta _a \tau _a r(t). \end{aligned}$$

This can be rewritten in terms of the time-dependent deviations from the new steady state as follows

9a $$\begin{aligned} \tau _m \frac{d}{dt} (\langle v(t) \rangle - \langle v \rangle _\varepsilon ) =&- (\langle v(t) \rangle - \langle v \rangle _\varepsilon ) - (\langle a(t) \rangle - \langle a \rangle _\varepsilon ) \nonumber \\&-\tau _m (v_T - v_R) (r(t)-r_\varepsilon ) \end{aligned}$$

9b $$\begin{aligned} \tau _a \frac{d}{dt} (\langle a(t) \rangle {-} \langle a \rangle _\varepsilon ) =&- (\langle a(t) \rangle {-} \langle a \rangle _\varepsilon ) {+} \Delta _a \tau _a (r(t){-}r_\varepsilon ) \end{aligned}$$

To analyze the system’s post-stimulation dynamics, we employ the Laplace transform to convert the differential equations into algebraic equations in the Laplace domain, yielding

10a $$\begin{aligned} \tau _m (\left\langle \delta v \right\rangle + \lambda {\hat{v}}(\lambda ))&= - {\hat{v}}(\lambda ) - {\hat{a}}(\lambda ) - \tau _m (v_T - v_R) {\hat{r}}(\lambda ) \end{aligned}$$

10b $$\begin{aligned} \tau _a (\left\langle \delta a \right\rangle + \lambda {\hat{a}}(\lambda ))&= - {\hat{a}}(\lambda ) + \Delta _a \tau _a {\hat{r}}(\lambda ). \end{aligned}$$

Here, ${\hat{v}}(\lambda )$, ${\hat{a}}(\lambda )$, and ${\hat{r}}(\lambda )$ denote the Laplace transforms of the deviations from post-stimulation steady-state for mean membrane potential, mean adaptation, and firing rate, respectively (we omit averaging brackets for the ease of notation). Numerically we compute the Laplace transforms starting from the stimulus onset $t_\varepsilon$ over a sufficiently long duration $T_{\mathscr {L}}$ as follows

11a $$\begin{aligned} {\hat{v}}(\lambda )&= \int _{t_\varepsilon }^{t_\varepsilon + T_{\mathscr {L}}} dt e^{-\lambda t} (\langle v(t) \rangle - \langle v \rangle _\varepsilon ) \end{aligned}$$

11b $$\begin{aligned} {\hat{a}}(\lambda )&= \int _{t_\varepsilon }^{t_\varepsilon + T_{\mathscr {L}}} dt e^{-\lambda t} (\langle a(t) \rangle - \langle a \rangle _\varepsilon )\end{aligned}$$

11c $$\begin{aligned} {\hat{r}}(\lambda )&= \int _{t_\varepsilon }^{t_\varepsilon +T_{\mathscr {L}}} dt e^{-\lambda t} (r(t) - r_\varepsilon ) . \end{aligned}$$

**Fig. 3:**
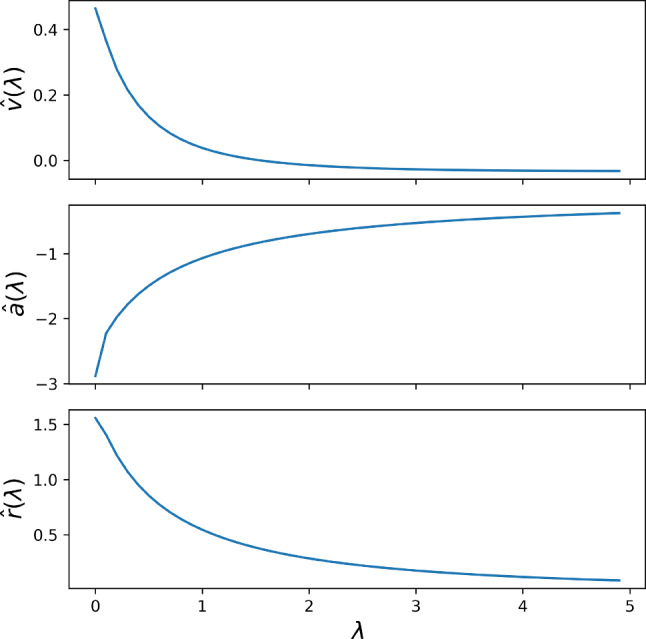
Laplace transforms of the time-dependent averages of membrane potential, adaptation variable, and firing rate minus their steady-state averages after stimulus onset (Eqs. (11a)–(11c)) for the data shown in Fig. 2

In Fig. 2 it can be seen that within the chosen time window $T_{\mathscr {L}}$ (light gray area) the mean values approach their steady state values very closely, thus the infinite limit of the true Laplace transform can be well approximated (Fig. 3).

Rearranging Eq. (10a) allows for the expression of the Laplace transform of the adaptation variable, ${\hat{a}}(\lambda )$, as follows

12 $$\begin{aligned} {\hat{a}}(\lambda )&= - \tau _m (\left\langle \delta v \right\rangle + \lambda {\hat{v}}(\lambda ) + (v_T - v_R) {\hat{r}}(\lambda )) - {\hat{v}}(\lambda ). \end{aligned}$$

Remarkably, ${\hat{a}}(\lambda )$ depends solely on the statistics of the membrane voltage, the firing rate, and the parameter $\tau _m$. The subsequent substitution of ${\hat{a}}(\lambda )$ and the expressions for $\left\langle \delta a \right\rangle$ in Eq. (6) and $\Delta _a$ in Eq. (7) into Eq. (10b) results in

13 $$\begin{aligned} \tau _a&= \frac{\tau _m h(\lambda ) + \delta r {\hat{v}}(\lambda ) + (\varepsilon - \left\langle \delta v \right\rangle ) \cdot {\hat{r}}(\lambda )}{\delta r \cdot (\varepsilon - \left\langle \delta v \right\rangle - \tau _m g(\lambda ) - \lambda {\hat{v}}(\lambda ))}. \end{aligned}$$

Here, $h(\lambda )$ and $g(\lambda )$ are defined by

14 $$\begin{aligned} h(\lambda )&= \delta r \left\langle \delta v \right\rangle + \delta r \lambda {\hat{v}}(\lambda ), \end{aligned}$$

15 $$\begin{aligned} g(\lambda )&= (v_T - v_R) \delta r + \lambda (\left\langle \delta v \right\rangle + \lambda {\hat{v}}(\lambda ) + (v_T - v_R) {\hat{r}}(\lambda )). \end{aligned}$$

We would like to point out that we know these functions once we have computed the Laplace transforms of the voltage and the firing rate and their steady-state values.

In the following, we exploit the fact that this equation holds for all $\lambda \ge 0$. Inserting two values, $\lambda _1$ and $\lambda _2$, yields a quadratic equation, which can be solved for $\tau _m$:

16 $$\begin{aligned} \tau _{m 1,2} =&\pm \frac{1}{2(g_1 h_2 - g_2 h_1)} \bigg [ \biggl ( - 4 (g_1 h_2 - g_2 h_1)\nonumber \\&(\lambda _1 {\hat{r}}_2 \varepsilon {\hat{v}}_1 - \lambda _2 {\hat{r}}_1 \varepsilon {\hat{v}}_2 - \lambda _1 {\hat{r}}_2 {\hat{v}}_1 \left\langle \delta v \right\rangle \nonumber \\&+ \lambda _2 {\hat{r}}_1 {\hat{v}}_2 \left\langle \delta v \right\rangle + \lambda _1 \delta r {\hat{v}}_1 {\hat{v}}_2 - \lambda _2 \delta r {\hat{v}}_1 {\hat{v}}_2 \nonumber \\&+ {\hat{r}}_1 \varepsilon ^2 - {\hat{r}}_2 \varepsilon ^2 - 2 {\hat{r}}_1 \varepsilon \left\langle \delta v \right\rangle + 2 {\hat{r}}_2 \varepsilon \left\langle \delta v \right\rangle + {\hat{r}}_1 \left\langle \delta v \right\rangle ^2 \nonumber \\&- {\hat{r}}_2 \left\langle \delta v \right\rangle ^2 + \delta r \varepsilon {\hat{v}}_1 - \delta r \varepsilon {\hat{v}}_2 - \delta r {\hat{v}}_1 \left\langle \delta v \right\rangle + \delta r {\hat{v}}_2 \left\langle \delta v \right\rangle ) \nonumber \\&+ (- g_2 {\hat{r}}_1 \varepsilon + g_1 {\hat{r}}_2 \varepsilon + g_2 {\hat{r}}_1 \left\langle \delta v \right\rangle - g_1 {\hat{r}}_2 \left\langle \delta v \right\rangle - g_2 \delta r {\hat{v}}_1\nonumber \\&+ g_1 \delta r {\hat{v}}_2 - h_1 \lambda _2 {\hat{v}}_2 \nonumber \\&+ h_2 \lambda _1 {\hat{v}}_1 + h_1 \varepsilon - h_2 \varepsilon - h_1 \left\langle \delta v \right\rangle + h_2 \left\langle \delta v \right\rangle )^2 \biggl )^{\frac{1}{2}} \nonumber \\&+ g_2 {\hat{r}}_1 \varepsilon - g_1 {\hat{r}}_2 \varepsilon - g_2 {\hat{r}}_1 \left\langle \delta v \right\rangle + g_1 {\hat{r}}_2 \left\langle \delta v \right\rangle \nonumber \\&+ g_2 \delta r{\hat{v}}_1 - g_1 \delta r {\hat{v}}_2 + h_1 \lambda _2 {\hat{v}}_2\nonumber \\&- h_2 \lambda _1 {\hat{v}}_1 - h_1 \varepsilon + h_2 \varepsilon + h_1 \left\langle \delta v \right\rangle - h_2 \left\langle \delta v \right\rangle \bigg ] \end{aligned}$$

It turns out that only one of the two solutions is positive and therefore physically plausible:

17 $$\begin{aligned} \tau _{m}&= \frac{1}{2(g_1 h_2 - g_2 h_1)} \bigg [ \biggl ( - 4 (g_1 h_2 - g_2 h_1) (\lambda _1 {\hat{r}}_2 \varepsilon {\hat{v}}_1 \nonumber \\&- \lambda _2 {\hat{r}}_1 \varepsilon {\hat{v}}_2 - \lambda _1 {\hat{r}}_2 {\hat{v}}_1 \left\langle \delta v \right\rangle + \lambda _2 {\hat{r}}_1 {\hat{v}}_2 \left\langle \delta v \right\rangle + \lambda _1 \delta r {\hat{v}}_1 {\hat{v}}_2 \nonumber \\&- \lambda _2 \delta r {\hat{v}}_1 {\hat{v}}_2 + {\hat{r}}_1 \varepsilon ^2 - {\hat{r}}_2 \varepsilon ^2 - 2 {\hat{r}}_1 \varepsilon \left\langle \delta v \right\rangle \nonumber \\&+ 2 {\hat{r}}_2 \varepsilon \left\langle \delta v \right\rangle + {\hat{r}}_1 \left\langle \delta v \right\rangle ^2 - {\hat{r}}_2 \left\langle \delta v \right\rangle ^2 + \delta r \varepsilon {\hat{v}}_1\nonumber \\&- \delta r \varepsilon {\hat{v}}_2 - \delta r {\hat{v}}_1 \left\langle \delta v \right\rangle + \delta r {\hat{v}}_2 \left\langle \delta v \right\rangle ) \nonumber \\&+ (- g_2 {\hat{r}}_1 \varepsilon + g_1 {\hat{r}}_2 \varepsilon + g_2 {\hat{r}}_1 \left\langle \delta v \right\rangle - g_1 {\hat{r}}_2 \left\langle \delta v \right\rangle - g_2 \delta r {\hat{v}}_1 \nonumber \\&+ g_1 \delta r {\hat{v}}_2 - h_1 \lambda _2 {\hat{v}}_2 + h_2 \lambda _1 {\hat{v}}_1 + h_1 \varepsilon \nonumber \\&- h_2 \varepsilon - h_1 \left\langle \delta v \right\rangle + h_2 \left\langle \delta v \right\rangle )^2 \biggl )^{\frac{1}{2}} \nonumber \\&+ g_2 {\hat{r}}_1 \varepsilon - g_1 {\hat{r}}_2 \varepsilon - g_2 {\hat{r}}_1 \left\langle \delta v \right\rangle + g_1 {\hat{r}}_2 \left\langle \delta v \right\rangle + g_2 \delta r{\hat{v}}_1 \nonumber \\&- g_1 \delta r {\hat{v}}_2 + h_1 \lambda _2 {\hat{v}}_2 - h_2 \lambda _1 {\hat{v}}_1 - h_1 \varepsilon + h_2 \varepsilon \nonumber \\&+ h_1 \left\langle \delta v \right\rangle - h_2 \left\langle \delta v \right\rangle \bigg ] \end{aligned}$$

**Fig. 4:**
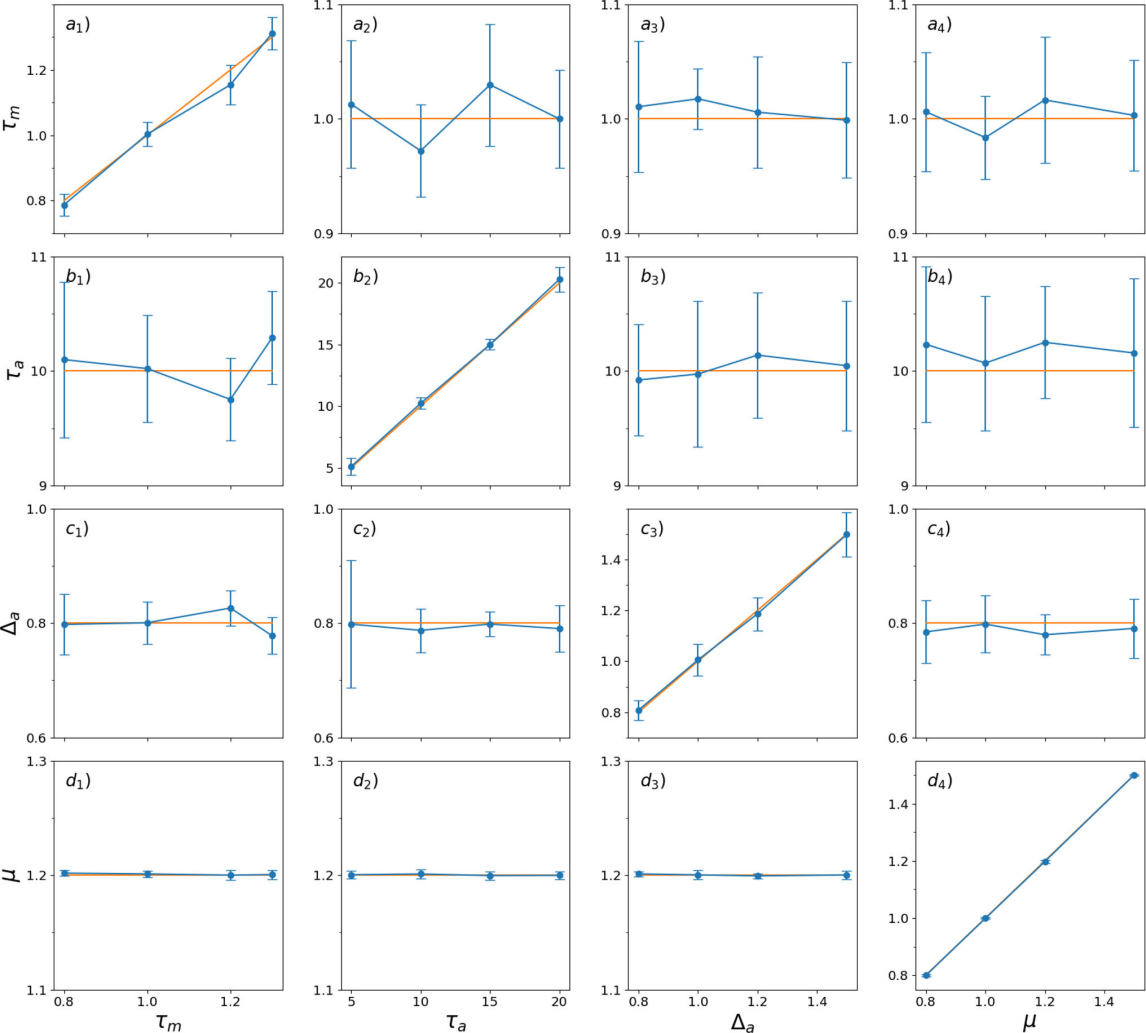
Estimated parameters $\tau _m$ (Eq. 17), $\tau _a$ (13), $\Delta _a$ (Eq. 7), and $\mu$ (Eq. 18) from 10 runs of $N_{\text {trials}}=1000$, each obtained from a simulation of Eqs. 1a–1c for a time window of $T=1000$ time units. Parameters are varied around the default values, $\tau _m = 1$, $\Delta _a = 0.8$, $\tau _a = 10$, $\tau _\eta = 1$, $\sigma ^2 = 1$, $s = 2.0$; The prescribed values are indicated by the orange lines; the estimated values are displayed by blue lines, symbols and error bars. The integration step for the simulation was $\Delta t = 10^{-5}$, saved with a time step of $\Delta t_s = 10^{-3}$

**Fig. 5:**
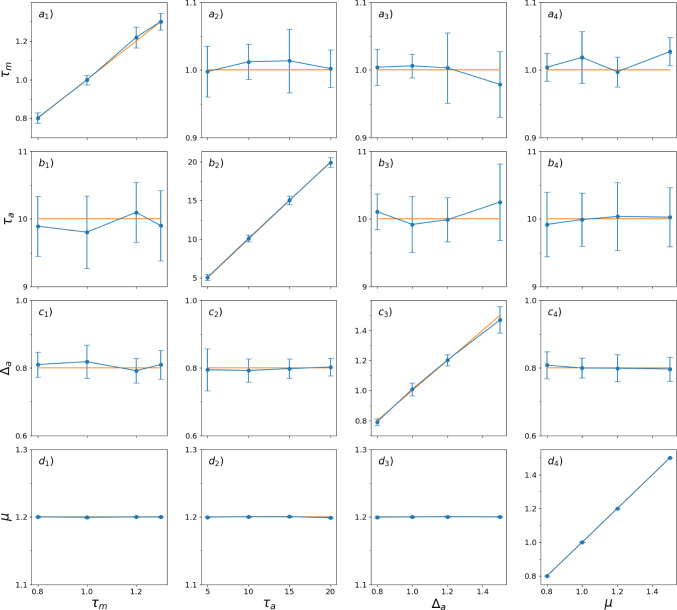
Estimated parameters $\tau _m$ (Eq. 17), $\tau _a$ (Eq. 13), $\Delta _a$ (Eq. 7), and $\mu$ (Eq. 18) from 10 runs of $N_{\text {trials}}=1000$, each obtained from a simulation of Eqs. 1a– 1c for a time window of $T=1000$ time units. Parameters are varied around the default values, $\tau _m = 1$, $\Delta _a = 0.8$, $\tau _a = 10$, $\tau _\eta = 0.1$, $\sigma ^2 = 1$, $s = 2.0$; The prescribed values are indicated by the orange lines; the estimated values are displayed by blue lines, symbols and error bars. The integration step for the simulation was $\Delta t = 10^{-5}$, saved with a time step of $\Delta t_s = 10^{-3}$

Importantly, the determination of $\tau _m$ relies solely on measurable quantities like membrane voltage *v*(*t*) and firing rate *r*(*t*), along with their Laplace transforms and on the amplitude $\varepsilon$ of the controlled stimulus current.

In the next step, the calculated value of $\tau _m$ is used to determine the adaptation time $\tau _a$ via Eq. (13). The results of both $\tau _a$ and $\tau _m$ allow the calculation of $\Delta _a$ via Eq. (7). Finally, the mean constant input current, $\mu$, can be computed directly from the steady-state Eqs. (3a and 3b) using the determined parameters

18 $$\begin{aligned} \mu = \langle v \rangle _0 + \Delta _a \tau _a r_0 + \tau _m (v_T - v_R) r_0. \end{aligned}$$

In conclusion, our analytical approach allows the determination of four key parameters of the adaptive integrate-and-fire model: $\tau _a$, $\Delta _a$, $\tau _m$, and $\mu$. In the next section, we will confirm our findings through stochastic simulations of the model. Here we aim to recover the prescribed values of the system parameters from a finite set of simulation trials.

## Validation of parameter estimation through numerical simulations

In the simulations, the four model parameters $\mu , \tau _m, \tau _a, \Delta _a$ are systematically varied around a default parameter set of $\mu =1.2$, $\tau _m = 1$, $\Delta _a = 0.8,$ and $\tau _a = 10$. In addition, we choose throughout a perturbation amplitude of $\varepsilon =2$ and the noise parameters such that the input fluctuations are strong ($\sigma ^2=1$) and significantly correlated ($\tau _\eta =1$, see Fig. 4) or almost uncorrelated ($\tau _\eta =0.1$, see Fig. 5). Testing different combinations of model parameters demonstrates the validity of the estimations across a spectrum of conditions for a modest number of 1000 trials.

In Fig. 4 we vary in the four columns the parameters $\tau _m, \tau _a, \Delta _a,$ and $\mu$, respectively. These variations respect a number of physiological boundary conditions: The adaptation time constant is significantly larger than the membrane time constant $\tau _m$, the effective input current $\mu$ is varied such that we explore both the excitable ($\mu <1$) and the mean-driven regime ($\mu >1$), which are both observed experimentally, and the adaptation strength $\Delta _a$ is varied such that the difference between the steady states are physiologically reasonable (Benda and Herz 2003). Because we vary only one parameter at a time, the other parameters should not change and be close to a horizontal line in the respective column, while the estimate of the changed parameter should fall on the identity line. Indeed, for all parameter variations, the estimated parameters are very close to the prescribed ones.

Of course, since we deal with a finite number of trials, the estimates exhibit some measurement noise. Interestingly, the random deviations in the estimates of $\tau _a$ and $\Delta _a$ obey an inverse relationship (Fig. 4b, c best visible in the first and last columns), arising from their reciprocal relationship in Eq. (7). On the contrary, the base current $\mu$ displays a particularly small measurement error across all parameter variations (Fig. 4d). When we decrease the correlation time of the noise by one order of magnitude (Fig. 5), our formulas for the extraction of the parameters still work, and the numerical error seems to be for most parameter variations even smaller than before. Similarly, we find that when the noise variance $\sigma ^2$ is decreased the method of parameter extraction still works very well (not shown).

**Fig. 6:**
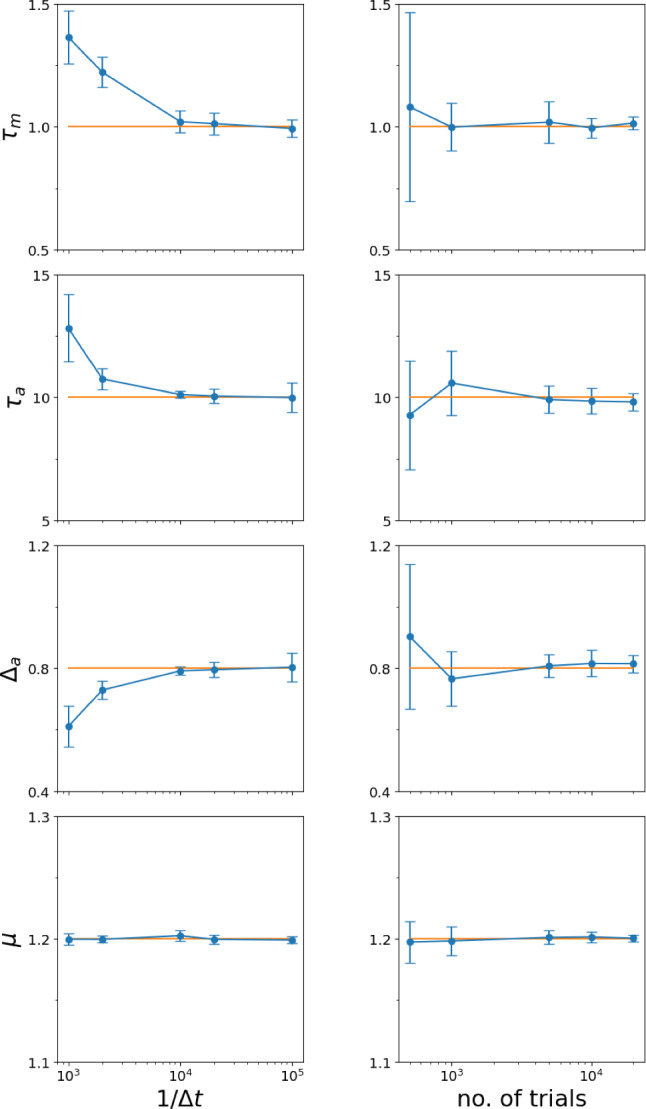
Estimated parameters $\tau _m$ (Eq. 17), $\tau _a$ (Eq. 13), $\Delta _a$ (Eq. 7), and $\mu$ (Eq. 18) from top to bottom versus inverse of the simulation time step $\Delta t$ (left) and versus the total number of simulation trials (right). Simulations were conducted with standard parameters ($\tau _m = 1$, $\tau _a = 10$, $\Delta _a = 0.8$, $\mu =1.2$, $\tau _\eta = 1$, $\sigma ^2 = 1$, $\varepsilon = 2$) and $N_{\text {trials}}=1000$ (left) and simulation time step $\Delta t = 10^{-5}$ (right). Yellow line: prescribed parameters

Because we use a finite number of stochastic simulations performed with a non-vanishing time step, we may expect systematic and random deviations from the true values of the parameters. How sensitive is our method to a change of the time step $\Delta t$ and the number of trials? These problems are inspected in Fig. 6. On the left we change the inverse of the simulation time step $\Delta t$ and find indeed systematic deviations for almost all parameters if the time step is too large ($\Delta t > 10^{-4}$). Starting with values around $\Delta t = 10^{-4}$ we find a convergence to the prescribed values, i.e. the systematic errors become significantly smaller than the random deviations (indicated by the error bars). Increasing the number of trials (Fig. 6, right) entails the predictable consequence, that all error bars are drastically reduced. There are no systematic deviations visible as we have used a sufficiently small time step.

## Summary

We have successfully derived explicit analytical formulas to extract parameters from a stochastic integrate-and-fire model with adaptation, using ensemble averages of membrane voltage and spike trains in response to a current step. We have determined four key parameters: the mean input current, the membrane time constant, the adaptation time constant, and the adaptation strength.

Unlike previous methods of parameter extraction that require the knowledge of the noisy input current (Badel et al. 2008a) or fit procedures (see e.g. Brette and Gerstner 2005; Teeter et al. 2018), our approach only needs the voltage trace in response to a current step to estimate parameters. Importantly, it works well even when only a few data sets are available, making it very suitable for experiments where data may be sparse. The formulas we have developed could be directly applied to experimental data to determine if the adaptive integrate-and-fire model accurately reflects actual neural dynamics. This method could also help identifying neuron types based on their firing and adaptation characteristics (Teeter et al. 2018).

For the estimation of the membrane time constant but also for the extraction of the noise parameters $\sigma$ and $\tau _\eta$ (a problem that we did not address here) a simple alternative approach exists that is also often applied in experiments. A strongly hyperpolarizing current will prevent firing, thus eliminating spike-triggered adaptation, and hence analysis of the temporal correlations of the spike-free voltage trace will betray the membrane time constant and the parameters of the intrinsic noise. Unfortunately, all these parameters may also be effectively changed upon strong hyperpolarization because for instance the kinetics of channel noise depends strongly on the holding potential of the cell. It would be nevertheless instructive to see how the membrane time constant and mean input determined by our and by the hyperpolarization methods compare. We also note that without hyperpolarization information on hidden parameters may be extracted by the voltage trace following a spike: the change in slope of the voltage over time may provide estimates of the hidden adaptation variable, and also the mean time course of the voltage is certainly affected by both the membrane time constant and the adaptation time constant. A future study providing a quantitative comparison of the different ways to extract parameters from limited data is certainly desirable.

Our approach may also help to experimentally test the fluctuation-response relations put forward by Lindner (2022). Exactly for the model inspected in our paper, the following relation should hold true:

19 $$\begin{aligned} \chi _x=\frac{ \left( v_T-v_R+\frac{\Delta _a \tau _a}{1+i\omega \tau _a}\right) S_{xx}+ (1+i\omega \tau _m) S_{xv}}{S_{\eta \eta }} \end{aligned}$$

Here we have on the left hand side the susceptibility, which is the Fourier transform of the linear-response function relating the time-dependent modulation of the instantaneous firing rate to a weak current stimulus (see e.g. Knight 1972; Lindner 2002; Fourcaud and Brunel 2002). On the right hand side we have stationary statistics without stimulus in the numerator and the intrinsic noise spectrum in the denominator. The latter is generally unknown, whereas both the spontaneous spike statistics and the response to an external stimulus can be measured. Knowing by our method all the parameters of the neuron (appearing in the numerator on the right-hand side of Eq. (19), we can now use Eq. (19) to determine the noise spectrum. We would like to emphasize that the noise process does not have to be an Ornstein-Uhlenbeck process - any Gaussian noise with vanishing mean value will do. This was already tested in Lindner (2022) for a noise consisting of the superposition of two independent Ornstein-Uhlenbeck processes with distinct correlation times and a narrow-band noise. Assuming the parameters of the hidden adaptation variable as known, the intrinsic noise spectrum could be successfully recovered (see Fig. 2b in Lindner (2022)). The contribution of our paper is to make the parameters needed in the above relation available from step-current experiments.

An interesting challenge is to extend our approach to neuron models that incorporate more realistic features, such as subthreshold nonlinearities as in the exponential integrate-and-fire model (Fourcaud-Trocmé et al. 2003), the neural refractory period (Berry and Meister 1998; Puttkammer and Lindner 2024), power-law adaptation (Pozzorini et al. 2013), or conductance noise (Richardson and Gerstner 2005; Lindner and Longtin 2006; Wolff and Lindner 2008; Richardson 2024). In many if not all of these cases, the formal linearity of the problem that allowed for a simple solution of the problem is, unfortunately, gone. It remains to be seen what kind of approximations can be worked out in these cases in order to still reliably determine the neural parameters. In conclusion, there are still a number of exciting research problems left to extend the approach for analytical parameter extraction in various directions.

## References

[CR1] Badel L, Lefort S, Brette R, Petersen CC, Gerstner W, Richardson MJ (2008a) Extracting non-linear integrate-and-fire models from experimental data using dynamic I-V curves. J Neurophysiol 99:656 19011924 10.1007/s00422-008-0259-4 PMC2798053

[CR2] Badel L, Lefort S, Berger TK, Petersen CCH, Gerstner W, Richardson MJE (2008b) Extracting non-linear integrate-and-fire models from experimental data using dynamic I-V curves. Biol Cybern 99:361 19011924 10.1007/s00422-008-0259-4 PMC2798053

[CR3] Benda J, Herz AVM (2003) A universal model for spike-frequency adaptation. Neural Comput 15:2523 14577853 10.1162/089976603322385063

[CR4] Berry M, Meister M (1998) Refractoriness and neural precision. J Neurosci 18:2200 9482804 10.1523/JNEUROSCI.18-06-02200.1998 PMC6792934

[CR5] Brette R, Gerstner W (2005) Adaptive exponential integrate-and-fire model as an effective description of neuronal activity. J Neurophysiol 94:3637 16014787 10.1152/jn.00686.2005

[CR6] Brunel N (2000) Dynamics of sparsely connected networks of excitatory and inhibitory spiking neurons. J Comput Neurosci 8:183 10809012 10.1023/a:1008925309027

[CR7] Brunel N, Sergi S (1998) Firing frequency of leaky integrate-and-fire neurons with synaptic current dynamics. J Theor Biol 195:87 9802952 10.1006/jtbi.1998.0782

[CR8] Brunel N, Chance FS, Fourcaud N, Abbott LF (2001) Effects of synaptic noise and filtering on the frequency response of spiking neurons. Phys Rev Lett 86:2186 11289886 10.1103/PhysRevLett.86.2186

[CR9] Burkitt AN (2006) A review of the integrate-and-fire neuron model: II. Inhomogeneous synaptic input and network properties. Biol Cyber 95:97 10.1007/s00422-006-0082-8 16821035

[CR10] Burkitt AN (2006) A Review of the Integrate-and-fire Neuron Model: I. Homogeneous Synaptic Input. Biol Cyber 95:1 10.1007/s00422-006-0068-6 16622699

[CR11] Campbell SR, Wang DL, Jayaprakash C (1999) Synchrony and desynchrony in integrate-and-fire oscillators. Neural Comput 11:1595 10490940 10.1162/089976699300016160

[CR12] de la Rocha J, Doiron B, Shea-Brown E, Josic K, Reyes A (2007) Correlation between neural spike trains increases with firing rate. Nature 448:802 17700699 10.1038/nature06028

[CR13] Droste F, Lindner B (2014) Integrate-and-fire neurons driven by asymmetric dichotomous noise. Biol Cybern 108:825 25037240 10.1007/s00422-014-0621-7

[CR14] Droste F, Lindner B (2017) Exact analytical results for integrate-and-fire neurons driven by excitatory shot noise. J Comp Neurosci 43:81 10.1007/s10827-017-0649-5 28585050

[CR15] Fisch K, Schwalger T, Lindner B, Herz A, Benda J (2012) Channel noise from both slow adaptation currents and fast currents is required to explain spike-response variability in a sensory neuron. J Neurosci 32:17332 23197724 10.1523/JNEUROSCI.6231-11.2012 PMC6621841

[CR16] Fourcaud N, Brunel N (2002) Dynamics of the firing probability of noisy integrate-and-fire neurons. Neural Comput 14:2057 12184844 10.1162/089976602320264015

[CR17] Fourcaud-Trocmé N, Hansel D, van Vreeswijk C, Brunel N (2003) How spike generation mechanisms determine the neuronal response to fluctuating inputs. J Neurosci 23:11628 14684865 10.1523/JNEUROSCI.23-37-11628.2003 PMC6740955

[CR18] Friedrich P, Vella M, Gulyás AI, Freund TF, Káli S (2014) A flexible, interactive software tool for fitting the parameters of neuronal models. Front Neuroinform 8:63 25071540 10.3389/fninf.2014.00063 PMC4091312

[CR19] Gerstner W, Naud R (2009) How good are neuron models? Science 326:379 19833951 10.1126/science.1181936

[CR20] Gerstner W, Kistler WM, Naud R, Paninski L (2014) Neuronal dynamics from single neurons to networks and models of cognition. Cambridge University Press, Cambridge

[CR21] Holden AV (1976) Models Stochastic Activity Neurones. Springer-Verlag, Berlin

[CR22] Huys QJ, Ahrens MB, Paninski L (2006) Efficient estimation of detailed single-neuron models. J Neurophysiol 96:872 16624998 10.1152/jn.00079.2006

[CR23] Iolov A, Ditlevsen S, Longtin A (2017) Optimal design for estimation in diffusion processes from first hitting times. SIAM-ASA J Uncertain 5:88

[CR24] Johannesma PIM (1968) Neural Networks. Springer, Berlin

[CR25] Knight BW (1972) Relationship between firing rate of a single neuron and level of activity in a population of neurons - experimental evidence for resonant enhancement in population response. J Gen Physiol 59:767 5025749 10.1085/jgp.59.6.767 PMC2203205

[CR26] Ladenbauer J, McKenzie S, English DF, Hagens O, Ostojic S (2019) Inferring and validating mechanistic models of neural microcircuits based on spike-train data. Nat Commun 10:4933 31666513 10.1038/s41467-019-12572-0 PMC6821748

[CR27] Lapicque L (1907) Recherches quantitatives sur l’excitation électrique des nerfs traitéecomme une polarization. J Physiol Pathol Gen 9:620

[CR28] Lindner B (2022b) arXiv2304.07027 [physics.bio-ph]

[CR29] Lindner B (2002) Coherence and stochastic resonance in nonlinear dynamical systems. Logos-Verlag, Berlin

[CR30] Lindner B (2022) A self-consistent analytical theory for rotator networks under stochastic forcing: effects of intrinsic noise and common input. Phys Rev Lett 129:198101 35778158 10.1063/5.0096000

[CR31] Lindner B, Longtin A (2006) Comment on “Characterization of Subthreshold Voltage Fluctuations in Neuronal Membranes’’ by M. Rudolph and A. Destexhe. Neural Comput 18:1896 16771657 10.1162/neco.2006.18.8.1896

[CR32] Lindner B, Doiron B, Longtin A (2005) Theory of oscillatory firing induced by spatially correlated noise and delayed inhibitory feedback. Phys Rev E 72:061919 10.1103/PhysRevE.72.061919 16485986

[CR33] Litwin-Kumar A, Doiron B (2012) Slow dynamics and high variability in balanced cortical networks with clustered connections. Nat Neurosci 15:1498 23001062 10.1038/nn.3220 PMC4106684

[CR34] Mankin R, Lumi N (2016) Statistics of a leaky integrate-and-fire model of neurons driven by dichotomous noise. Phys Rev E 93:052143 27300865 10.1103/PhysRevE.93.052143

[CR35] Moreno-Bote R, Parga N (2010) Response of integrate-and-fire neurons to noisy inputs filtered by synapses with arbitrary timescales: firing rate and correlations. Neural Comput 22:1528 20100073 10.1162/neco.2010.06-09-1036

[CR36] Paninski L, Simoncelli E, Pillow J (2003) Maximum likelihood estimation of a stochastic integrate-and-fire neural model. Adv Neural Inf Process Syst 16:1 10.1162/0899766042321797 15516273

[CR37] Pozzorini C, Naud R, Mensi S, Gerstner W (2013) Temporal whitening by power-law adaptation in neocortical neurons. Nat Neurosci 16:942 23749146 10.1038/nn.3431

[CR38] Puttkammer F, Lindner B (2024) Fluctuation-response relations for integrate-and-fire models with an absolute refractory period. Biol Cybern 118:1–13 38261004 10.1007/s00422-023-00982-9 PMC11068698

[CR39] Ricciardi LM (1977) Diffusion Processes and Related Topics on Biology. Springer-Verlag, Berlin

[CR40] Richardson MJE (2024) Linear and nonlinear integrate-and-fire neurons driven by synapticshot noise with reversal potentials. Phys Rev E 109:024407 38491664 10.1103/PhysRevE.109.024407

[CR41] Richardson MJE, Gerstner W (2005) Synaptic shot noise and conductance fluctuations affect the membrane voltage with equal significance. Neural Comput 17:923 10.1162/0899766053429444 15829095

[CR42] Richardson MJE, Swarbrick R (2010) Firing-rate response of a neuron receiving excitatory and inhibitory synaptic shot noise. Phys Rev Lett 105:178102 21231083 10.1103/PhysRevLett.105.178102

[CR43] Rossant C, Goodman D, Fontaine B, Platkiewicz J, Magnusson A, Brette R (2011) Fitting neuron models to spike trains. Front Neurosci 5:9 21415925 10.3389/fnins.2011.00009 PMC3051271

[CR44] Schwalger T, Fisch K, Benda J, Lindner B (2010) How noisy adaptation of neurons shapes interspike interval histograms and correlations. PLoS Comp Biol 6:e1001026 10.1371/journal.pcbi.1001026 PMC3002986 21187900

[CR45] Stein RB (1967) Some models of neuronal variability. Biophys J 7:37 19210981 10.1016/S0006-3495(67)86574-3 PMC1368056

[CR46] Teeter C, Iyern R, Menon V, Gouwens N, Feng D, Berg J, Szafer A, Cain N, Zeng H, Hawrylycz M (2018) Generalized leaky integrate-and-fire models classify multiple neuron types. Nat Commun 9:709 29459723 10.1038/s41467-017-02717-4 PMC5818568

[CR47] Teeter C, Iyer R, Menon V, Gouwens N, Feng D, Berg J, Szafer A, Cain N, Zeng H, Hawrylycz M et al (2018) Generalized leaky integrate-and-fire models classify multiple neuron types. Nat Commun 9:709 29459723 10.1038/s41467-017-02717-4 PMC5818568

[CR48] Treves A (1993) Mean-field analysis of neuronal spike dynamics. Netw Comput Neural Syst 4:259

[CR49] Tuckwell HC (1988) Introduction to Theoretical Neurobiology. Cambridge University Press, Cambridge

[CR50] Tuckwell HC (1989) Stochastic Processes in the Neuroscience. SIAM, Philadelphia

[CR51] Uhlenbeck GE, Ornstein LS (1930) On the theory of the Brownian motion. Phys Rev 36:823

[CR52] Vilela RD, Lindner B (2009) Are the input parameters of white-noise-driven integrate & fire neurons uniquely determined by rate and CV? J Theor Biol 257:90 19063904 10.1016/j.jtbi.2008.11.004

[CR53] Vilela RD, Lindner B (2009) A comparative study of three different integrate-and-fire neurons: spontaneous activity, dynamical response, and stimulus-induced correlation. Phys Rev E 80:031909 10.1103/PhysRevE.80.031909 19905148

[CR54] Wolff L, Lindner B (2008) A method to calculate the moments of the membrane voltage in a model neuron driven by multiplicative filtered shot noise. Phys Rev E 77:041913 10.1103/PhysRevE.77.041913 18517662

